# Innovating neurosurgical training: a comprehensive evaluation of a 3D-printed intraventricular neuroendoscopy simulator and systematic review of the literature

**DOI:** 10.3389/fsurg.2024.1446067

**Published:** 2024-11-05

**Authors:** Attill Saemann, Adriana De Rosa, Jokin Zubizarreta Oteiza, Neha Sharma, Florian M. Thieringer, Jehuda Soleman, Raphael Guzman

**Affiliations:** ^1^ Department of Neurosurgery, University Hospital Basel, Basel, Switzerland; ^2^ Department of Oral and Cranio-Maxillofacial Surgery and 3D Print Lab, University Hospital Basel, Basel, Switzerland; ^3^ Medical Additive Manufacturing Research Group (Swiss MAM), Department of Biomedical Engineering, University of Basel, Basel, Switzerland; ^4^ Faculty of Medicine, University of Basel, Basel, Switzerland

**Keywords:** neuroendoscopy, neurosurgery, simulation training, three-dimensional printing, surgical training

## Abstract

**Objective:**

The objective of this study was to develop and evaluate a low-cost 3D-printed simulator to improve the ability of neurosurgical residents to handle and coordinate endoscopes in performing technically demanding procedures such as neuroendoscopic removal of ventricular tumors or endoscopic third ventriculostomy (ETV).

**Methods:**

The simulator was developed, printed in-house, and evaluated in a trial involving neurosurgery residents who performed ETV and intraventricular tumor resection tasks using it. Participants completed a questionnaire that assessed various aspects of the simulator's effectiveness, including anatomical visualization, procedural understanding, competency enhancement, and subjective impressions.

**Results:**

A total of 12 participants were included in the evaluation. The majority (*n* = 7, 53.85%) were male, with a mean age of 29.8 ± 3.27 years and 4 ± 2 years of neurosurgical experience. All participants agreed or strongly agreed (4.5 ± 0.50) that the 3D printed simulator helped develop systematic intraventricular visualization and understanding of surgical steps (4.42 ± 0.64). The handling of the endoscope was rated as realistic (4.5 ± 0.50), while the haptic qualities of the tumor were rated lower (3.83 ± 0.80; 3.92 ± 0.64). Training increased competence (4.25 ± 0.45) and coordination skills (4.5 ± 0.50), with 75% (*n* = 9) feeling more confident with neuroendoscopic instruments and 91.7% (*n* = 11) in future procedures.

**Conclusion:**

The developed 3D-printed simulator offers an accessible and practical training resource for neurosurgical residents, addressing the limitations of traditional training methods. The simulator appears to improve procedural skills and the competence of future neurosurgeons, potentially improving patient safety and outcomes in neurosurgical practice.

## 1 Introduction

Manual training to refine technical skills remains critical to training and education in all surgical specialties (1). Performing technically demanding procedures at a high level of safety is a crucial element of neurosurgical operations. Since the caseload in a subspecialized surgical discipline may be relatively low depending on the catchment area of the medical institution, providing a safe and effective training opportunity for neurosurgical residents is a focal point of state-of-the-art teaching hospitals.

The demand for the acquisition of advanced surgical skills is well established in neurosurgical training, necessitating extensive hands-on training and experience to overcome a steep learning curve (2–6) However, contemporary challenges have reduced operative exposure during residency, including resident work hours restrictions and increased efficiency of operation time (7–11). Consequently, there is excellent potential for simulation-based medical education (1, 2, 5, 12, 13).

This trend toward simulation training is pronounced in technically challenging neurosurgical procedures such as neuroendoscopic surgery, which involves intricate instrument manipulation, a potentially disorienting endoscopic environment, and poses distinct challenges due to the scarcity of practice cases (4–6). Additionally, mastering this method requires proficiency in ambidextrous manipulation, increased eye-hand coordination, and acclimatization to conceptualization in multiple dimensions (9). As traditional training models, such as cadavers and animal samples, face limitations in availability and ethical concerns, simulation-based tools, such as simulators and virtual reality (VR) systems, have emerged to address these challenges and improve the acquisition of specific clinical skills (7, 14–18).

Recent developments include advanced VR systems and 3D-printed anatomical models (12, 17, 18, 19). These simulation tools offer advantages such as a safe training environment, portability, reusability, and cost-effectiveness. However, they may need to fully replicate anatomical dissection's intricacies or complex procedures. In response to the need for effective neuroendoscopic training, we present a low-cost 3D-printed simulator developed and manufactured in-house. This simulator allows multiple intraventricular procedures such as neuroendoscopic removal of intraventricular tumors or endoscopic third ventriculostomy (ETV) and aims to improve endoscope handling skills and coordination.

This work aims to describe, compare, and validate our 3D-printed model and its benefits for educating young neurosurgeons. A systematic review was conducted to compare it with the current literature.

## 2 Methods

### 2.1 Developing the 3D model

Magnetic resonance imaging (MRI) (Philips Ingenia Elition X, Koninklijke Philips N.V., Amsterdam, the Netherlands) and computed tomography (CT) scans (Siemens SOMATOM, Siemens Healthcare GmbH, Erlangen, Germany) were combined to obtain the necessary data sets to design the neuroendoscopic training simulator. These datasets, sourced from the internal Picture Archiving and Communication System (PACS) database, were anonymized before being imported into medical image analysis software (Materialise Mimics v25.0, Materialise, Leuven, Belgium) using the Digital Imaging and Communications in Medicine (DICOM) format and then modified to the specific needs of simulation training. Therefore, no ethical consent was required for this study.

Radio-density-based segmentation used predetermined Hounsfield unit (HU) thresholds to delineate bone (226; 3,071) and soft tissues (−700; 225) to reconstruct the patient's skull and ventricles, respectively. Subsequently, both voxel-based models were transformed into 3D mesh representations and exported as Standard Tessellation Language (STL) files, serving as the foundation for simulator development.

To ensure the cost-effectiveness and flexibility of the training model, an emphasis was placed on making its components reusable and adaptable. Therefore, a modular approach was selected for the simulator design in computer-aided design (CAD) software (Geomagic Freeform Plus v2022.0.34, Oqton Inc., San Francisco, California, USA). The skull model was divided into three parts (top, middle, bottom), interconnected with protrusions and recesses for easy assembly postproduction. Furthermore, the shape of the ventricle model was modified to mimic the distended proportions seen in patients with hydrocephalus, facilitating realistic training scenarios.

One variant of the ventricle had openings in both the lateral ventricles and the third ventricle floor. These facilitated the insertion of a flexible membrane, allowing simulation of endoscopic fenestration during ETV and septostomy. The second version of the model, tailored for training in intraventricular tumor aspiration, omitted these openings so they could be filled with water for optimal tumor resection. Instead, it maintained open lateral ventricles for membrane placement, as in the first iteration. Furthermore, the patient's basilar artery was reconstructed to provide an additional anatomical reference point for surgical training.

**Figure 1:**
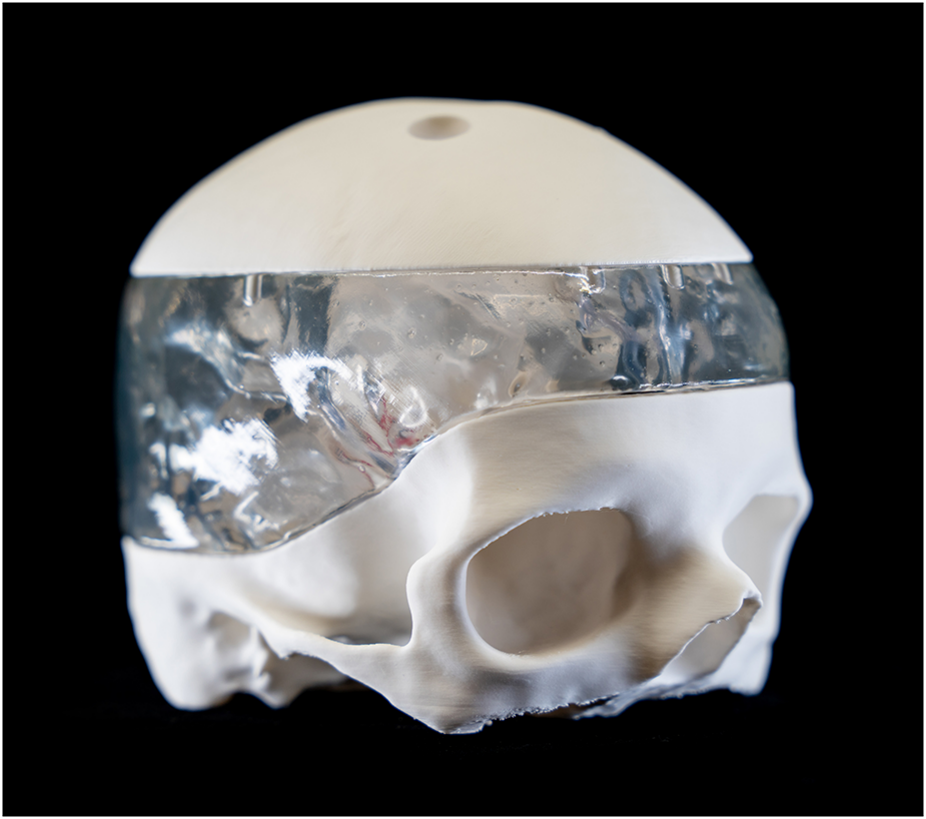
Lateral view of the 3D-printed model fully assembled.

**Figure 2:**
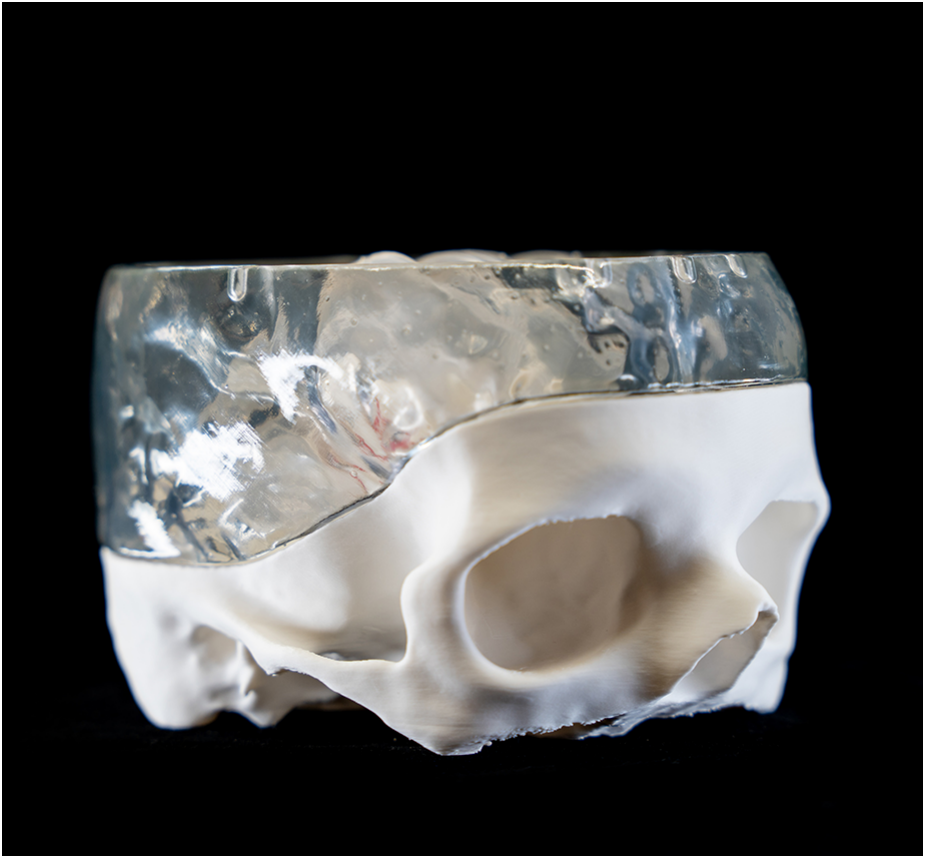
Lateral view of the 3D-printet model without the upper skull part.

**Figure 3:**
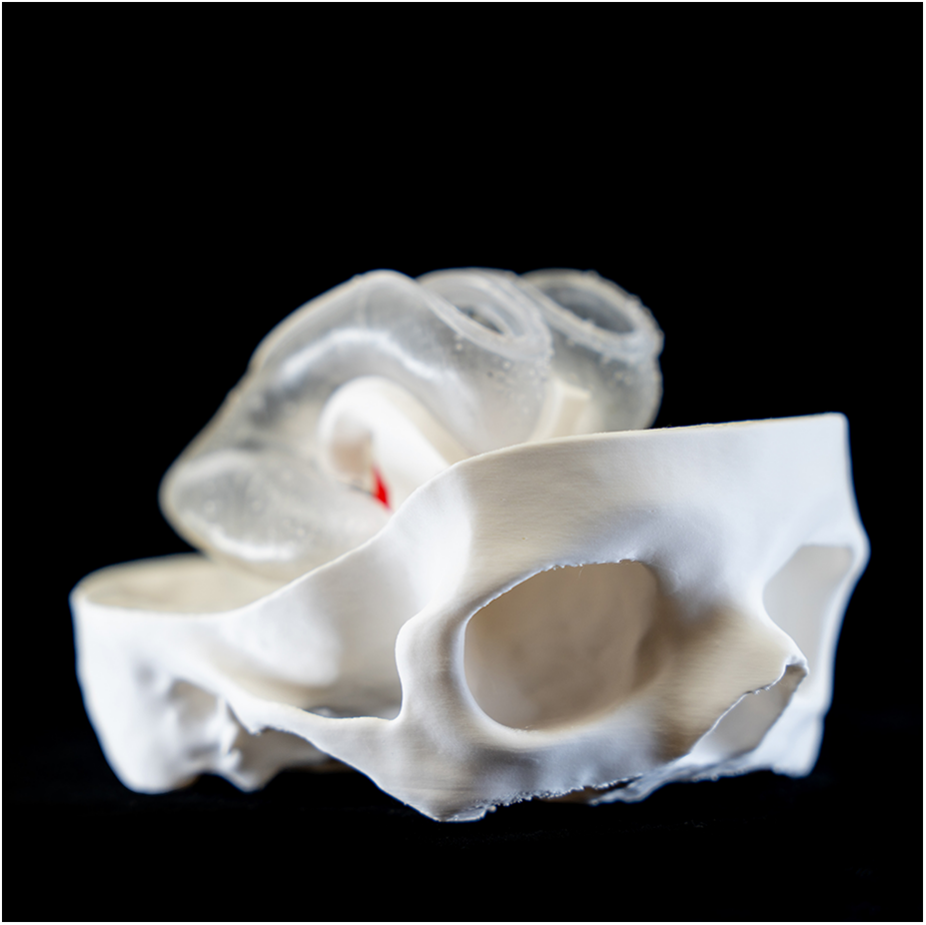
Lateral view of the ventricle model assembled in the lower part of the skull.

Two distinct additive manufacturing (AM) techniques were utilized for the in-house printing of models representing both hard and soft tissues. The upper and lower parts of the skull, together with the basilar artery, were printed using polylactic acid (PLA) filament (Bambu Lab PLA Matte, Bambulab, Shenzhen Tuozhu Technology Co., Ltd., Shenzhen, China) using a fused filament fabrication 3D printer (Bambu Lab X1-Carbon, Bambulab GmbH, Shenzhen, China). A proprietary slicing software (Bambu Studio v1.8.2, Bambulab GmbH, Shenzhen, China) was used to generate the necessary gcode files (Figures 1–3).

**Figure 4:**
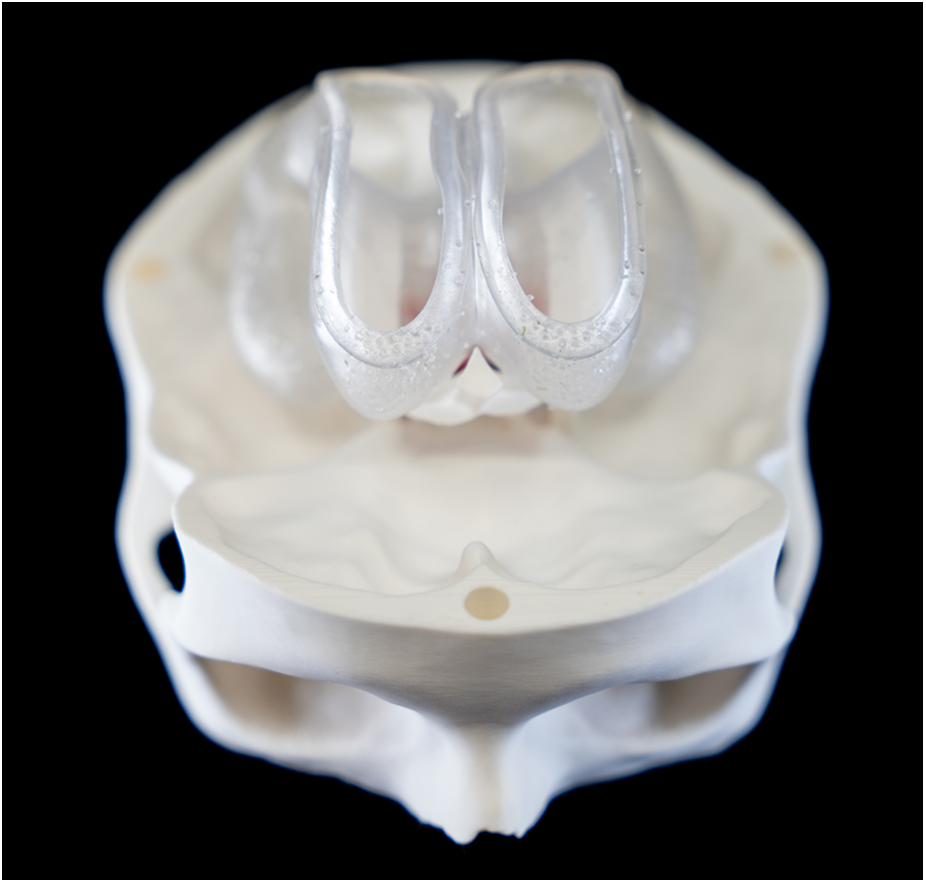
Frontal overview of the ventricle model.

**Figure 5:**
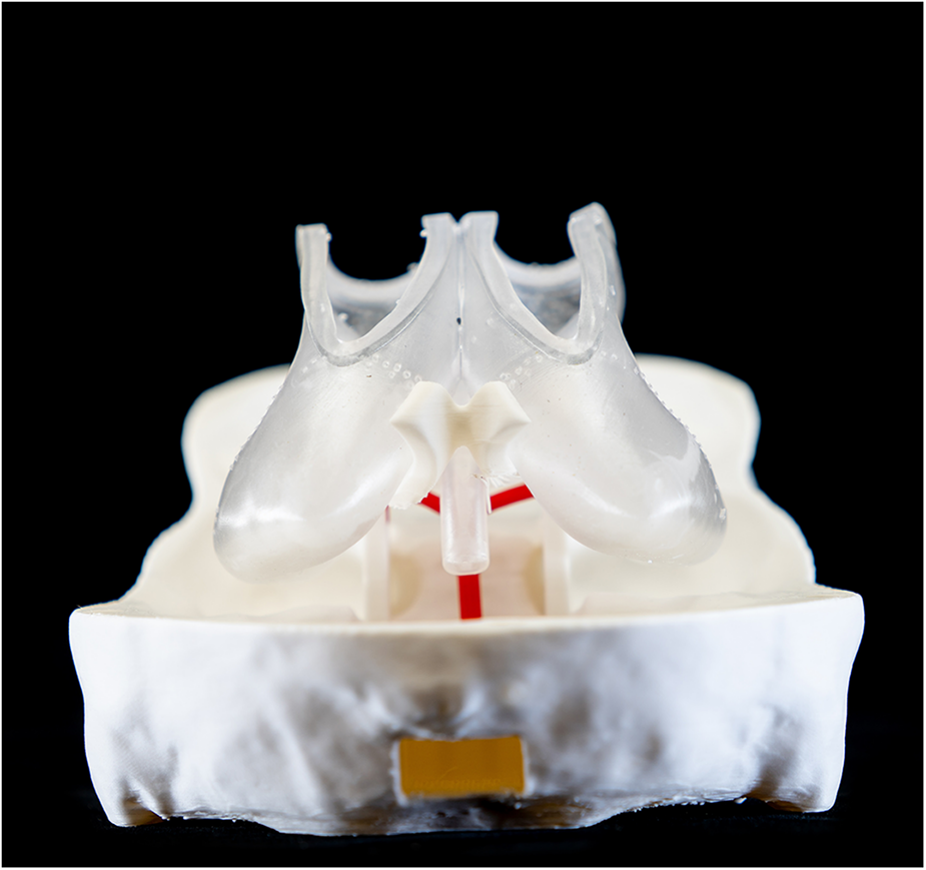
Occipital overview of the ventricle model and the basilary artery.

The ventricles were manufactured using a Stereolithography 3D printer (SLA) (Formlabs 3B, Formlabs Inc, Sommerville, Massachusetts, USA) and respective slicing software (Preform v.2.4.0-2216, Formlabs Inc, Sommerville, Massachusetts, USA). A soft and translucent elastomeric material (Elastic 50A resin V2, Formlabs Inc, Sommerville, MA, USA) was used to replicate the flexible nature of the tissue and enhance haptic perception during surgical training. Furthermore, the middle part of the skull model was manufactured in a rigid transparent material (Clear resin v4, Formlabs Inc, Sommerville, Massachusetts, USA) to optimize external visualization of the position of the endoscope tip positioning externally (Figures 4, 5). A gelatine layer could be placed on top of the ventricle, simulating the brain parenchyma when accessing the ventricle with the endoscope. Before assembly, all SLA 3D-printed parts were post-processed according to the manufacturer's guidelines (Supplementary Video S1).

### 2.2 Cost analysis and production time of the 3D-printed model

The skull model was 3D-printed with PLA filament and Clear resin with a fill density of 15%, which took approximately 24 h to print. Printing of the skull model consumed 795 g PLA and 250 ml of clear resin, corresponding to $77. By printing the middle part of the skull with PLA instead of Clear resin, the price can be reduced by $45. Elastasis 50A resin was used to print the ventricles, which took 22 h. It takes the same time to print 1 or 2 ventricles, as the number of layers stays the same when placing two models in the same printing bed. 120 ml of elastasis was consumed, corresponding to $35. In conclusion, the whole model costs either $67 or $112 depending on the materials used for the middle part of the skull model and can be printed in 24 h. However, these cost calculations only include raw material consumption, excluding the 3D printer, license fees, energy consumption, and costs of working time.

### 2.3 Study participants and evaluation

The study group that evaluated the 3D printed simulator consisted of neurosurgery residents from different departments in Switzerland with mixed levels of training. The trial was carried out during a neuroendoscopy course at the Institute of Anatomy of the University of Basel, Basel, Switzerland. Each participant performed an ETV and intraventricular tumor resection with the Söring Endoscopic Neurosurgical Pen (ENP, Söring GmbH, Quickborn, Germany) under the instruction and supervision of a senior board-certified neurosurgeon.

**Figure 6:**
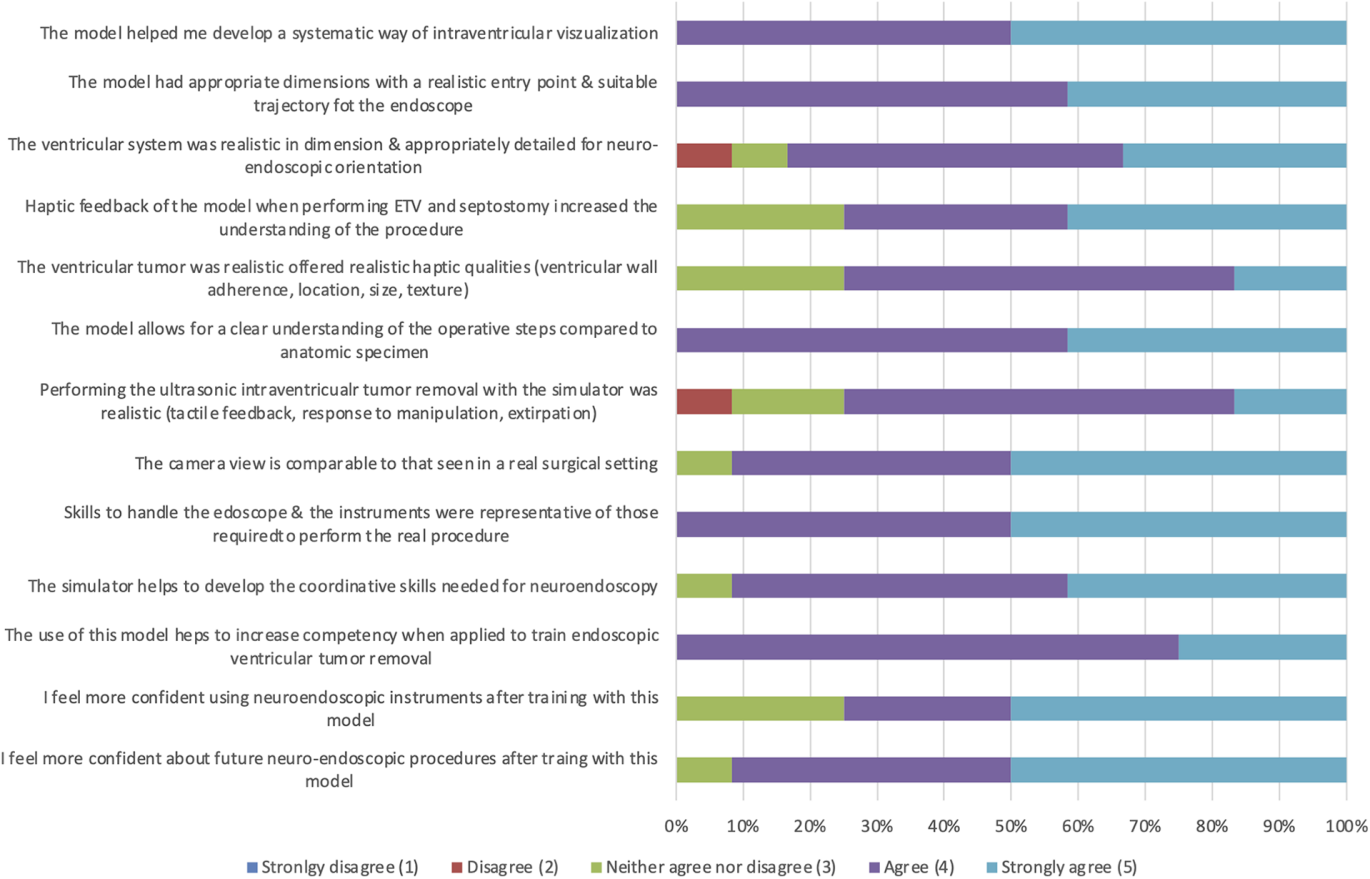
Results of the resident survey of the 3D printed neuro endoscopy model (*n* = 12).

Each participant followed the same surgical steps in the same case and model. Subsequently, a questionnaire was completed that contained demographic questions, questions about handling and executing the two tasks, and questions about anatomical orientation and subjective impressions of the model. The handling and teaching effectiveness of the 3D model were qualitatively assessed (Figure 6). Participants were asked to rate their level of agreement with each of the 12 questions using a 5-point Likert scale, with the following scores: 1, strongly disagree; 2, disagree; 3, neither agree nor disagree; 4, agree; and 5, strongly agree. It should be noted that a rating of 1 signified poor applicability, while a rating of 5 indicated excellent applicability of the model.

### 2.4 Literature review

The systematic review of the literature was conducted according to the Preferred Reporting Items for Systematic Reviews and Meta-Analyses (PRISMA) guidelines (20). PubMed and Embase databases were searched for relevant publications from 2010 to January 2024. For the database search, a search string around the following concepts was created: “neurosurgical procedures”, “neuroendoscope,” “ventriculostomy,” “simulation training”, “anatomic models,” and “three-dimensional printing”. All studies reporting neuroendoscopic intraventricular procedures performed on a 3D-printed model and in full-text English language were included. systematic reviews, case reports, and studies reporting intraventricular procedures that were not performed endoscopically and did not involve a 3D-printed model were excluded.

The study identification and selection process was carried out using cloud-based software (Rayyan—a web and mobile app for systematic reviews. Systematic Reviews 2016) and is summarized in the PRISMA flowchart (Supplementary Figure S1). Of the 388 identified publications, 89 were duplicates and 281 were excluded. Eighteen reports were sought for retrieval, 14 did not report the use of endoscopes or intraventricular application, and one was not a full-text article. Ultimately, three articles met our inclusion criteria.

In the systematic review, the included publications’ cohorts (10–20 participants) were primarily neurosurgical residents with varying experience levels. Each resident tested the model and completed a questionnaire for evaluation. Descriptive statistics were used to rate the model's appearance and the reproducibility of the procedure.

When looking at production design, time, and costs, the three different models were all designed through Digital Imaging and Communications in Medicine (DICOM) images, which had been converted and coded for 3D printing. Regarding production costs, Garling et al. proposed in 2018 a silicone-based ETV simulator and mimetic endoscope for $123, which can be produced in 48 h using free open-source software (9). In 2020, our group (Licci et al.) proposed a synthetic neuroendoscopic ultrasonic tumor removal simulator that can be printed in 4–7 h for $94 using open-source software. There is no information about the production time and cost of the simulator proposed by Weinstock et al. in 2017. The simulator has interchangeable plug-and-play components. They describe the cost as a study limitation and investigate the possibility of iterations with lower external fidelity to reduce production costs (8).

Both Licci et al. and Weinstock et al. focus on residents’ ratings and opinions to demonstrate the effectiveness of their models as innovative training and teaching tools for neuroendoscopic procedures. In contrast, Garling et al. focused on the model itself and described its production precisely and in detail, as well as the production of a mimetic endoscope. The survey in the study by Licci et al. also addresses the personal learning experience of the individual participants. 70% (*n* = 7) of the participants strongly agreed that this model improves proficiency when training individuals to remove ventricular tumors using endoscopic techniques (7).

In the study by Garling et al., the questionnaire contains mainly questions about the technical feasibility of the simulator and little about its teaching potential. However, 87% (*n* = 13) of the participants strongly agreed that the simulator was helpful in resident training (7–9). All surveyed agreed (mean Likert score 4.88) that the model effectively simulated the surgical procedure (8).

### 2.5 Statistical analysis

Descriptive statistics were conducted to summarize the ratings’ results. All results are given as mean scores with standard deviation and percentage. Statistical analysis was performed within the R database [R Core Team (2022). R: A language and environment for statistical computing, Vienna, Austria].

## 3 Results

### 3.1 Overall demographics

A total of 12 participants were included. Ten completed the questions about their sex, age, experience as a neurosurgical resident, and the caseload of the corresponding operations in real-life patients. The majority (*n* = 7, 53.85%) of the participants were men. The mean age was 29.8 ± 3.27 years. The average neurosurgical experience was 4 (±2) years, with an average lifetime caseload of 7.5 ± 3.74 cases. Two participants had never performed endoscopic neurosurgical procedures before (Table 1).

**Table 1:** Baseline characteristics of the participants.

Number of participants	12
Gender	
Male	7 (53.85%)
Female	2 (16.67%)
Non identified	3 (25%)
Mean age (years)	29.8 (± 3.27)
Neurosurgical experience (years)	4 (± 2)
Average lifetime caseload (cases)	7.5 (± 3.74)

### 3.2 Rating of the 3D printed simulator

All participants (*n* = 12) agreed or strongly agreed (4.5 ± 0.50) on the model, helping to develop a systematic way of intraventricular visualization and, consequently, a clear understanding of the individual steps of surgical procedures (4.42 ± 0.64). Furthermore, all rated endoscope handling is comparable to real surgical settings (4.5 ± 0.50). The questions about realistic haptic qualities of the ventricular tumor and its removal and mechanical properties were rated lowest (3.83 ± 0.80; 3.92 ± 0.64). However, all participants strongly agreed that training with this model helps increase competence in endoscopic ventricular tumor removal procedures (4.25 ± 0.45) and helps develop the coordination skills needed for neuroendoscopy (4.5 ± 0.50). Of the 12 participants, 75% (*n* = 9) felt more confident using neuroendoscopic instruments, while in 91.7% (*n* = 11) a stronger confidence for future neuroendoscopic procedures was described. The results of the questionnaires are summarized in Figure 6.

Tutors reported that the simulator's standardized anatomy facilitated a structured, step-by-step approach to teaching and assessing learning objectives. They noted that the ability to repeat specific parts of the procedure was beneficial in achieving proficiency in both dexterity and understanding. Additionally, the simplification of anatomy to essential reference structures improved orientation for both participants and instructors. This standardization enabled tutors to switch between different groups without having to adapt to new anatomical variations, thereby enhancing the consistency and efficiency of the training process.

## 4 Discussion

Our study introduces a cost-effective, in-house designed and 3D-printed reusable endoscopy simulator with a modular setup, built to enhance neurosurgical residents’ endoscope handling skills and coordination. Questionnaire-based evaluation revealed positive feedback on the effectiveness of the simulator in developing a systematic approach to intraventricular visualization, understanding surgical procedures, and increasing competency in endoscopic removal of ventricular tumors. Participants consistently rated the model well throughout our evaluation, indicating its effectiveness as a training tool. Our endoscopic training model is significantly more cost-effective than cadaver specimens. With a printing cost of $67, it is, to our knowledge, the most economical 3D-printed simulator for training neuroendoscopic procedures according to the current literature.

Traditional training models, such as cadavers and animal specimens, have limitations and ethical concerns for surgical training. First, the limited resemblance between animal and human brains undermines the reliability of animal models (7, 9). Furthermore, dedicated procedures like neuro-endoscopic training are hindered by the scarcity of cadaveric samples exhibiting the required anatomical changes, i.e., ventriculomegaly, thereby restricting their utility. Additionally, formaldehyde used for preservation purposes poses toxicity risks and can induce discomfort during practice sessions (7, 9). Regarding costs, a single human cadaveric head can vary from approximately $600 to $1,400 (21) while Mladina et al. describe a cost of $1,520 for a single resident to train (22). Furthermore, substantial expenses of maintaining an experimental laboratory *in vivo* must be considered (4, 7). Thus, due to their limited availability and applicability primarily to restricted training scenarios, they represent a costly and less accessible alternative. The minimal material expenses of our model offer a considerable advantage over cadaveric specimens. Additionally, since both the model and the digital data set can be reused, there is significant potential to reduce costs and time during production when reproducing the model. Moreover, the use of 3D-printed models proves advantageous in countries where laws restrict the use of cadavers in medical education and training programs (22).

When comparing our newly developed 3D-printed simulator to the previous version described by our group (Licci et al.) in 2020, multiple improvements were made based on the feedback received (7). A major upgrade in functionality is its multipurpose usage, so ETV, tumor aspiration, septostomy, and aequedoctoplasty can be performed on the same model when part of the modular build is exchanged. The adaptable components may be replaced individually, increasing efficiency since the large part of the simulator does not need to be replaced. By implementing new materials, the ventricle system could be flexibly printed and mimic its real-life elastic properties. Overall, we could reduce the total cost by $27 (29%) per model. When comparing the simulator evaluation, the overall feedback in both studies shows a high level of agreement across all elements, while, in our survey, there were disagreements about the quality of details for orientation and the haptic feedback when aspirating the tumor. Even though we used the same tumor models and the ventricle was printed with new and flexible materials, this feedback may indicate a grown expectation towards 3D-printed training models since the exposition to such models and their quality has increased strongly over the last years.

Different publications have shown the beneficial effect of 3D models on improving understanding of anatomy and surgical procedures (7–9, 11, 17–19). Recent developments in VR systems and 3D-printed anatomical models offer advantages such as a safe training environment, portability, reusability, and cost-effectiveness (7–12). Such models allow resident surgeons to perform repeatedly step-by-step procedures without concerns about patient safety. Moreover, a trainee can perform up to an entire basic procedure rather than incrementally acquiring it through training in a real patient, which can shorten the long learning curve (5, 6). VR simulators are recognized as effective technical skill training platforms, with systems now developed for endoscopic surgery. Although their initial maintenance costs are comparatively high among the different simulation-based training options, they offer the most visually comprehensive experience, superior to physical models, and have the advantage of simulating various procedures an infinite number of times at no additional cost (21). Some VR simulators also aim to include a real-time feedback system that provides information about instrument positions, force levels achieved, and trainee performance (5, 21). However, VR platforms usually lack tactile feedback compared to synthetic simulators. Second, the tools used during the simulation are not the actual operating instruments and do not allow realistic training like bimanual instrumentation (5). Therefore, 3D-printed synthetic simulators, including our model, excel in handling training instruments and procedural content. Additionally, Langridge et al. show that 3D printed models outperformed virtual 3D imaging and traditional 2D educational models regarding learning and comprehension. Their study revealed a statistically significant disparity in anatomy quiz scores between trainees who used 3D printed models and those who relied on 2D or 3D imaging for preparation (23). Both VR and 3D printed simulators have their benefits and disadvantages. The selection of the simulator should be based on specific learning objectives (5, 7, 21).

3D-printing technology is becoming an increasing part of medical education. Collaborative efforts, such as the exchange of knowledge and expertise among researchers and physicians, are crucial to advancing the development and use of simulation-based training tools around the world. While 15 years ago, the cost of a 3D printer ran from $10’000 to $500’000 and was restricted in accessibility, nowadays, this technology can be purchased by a broader group and can only cost as much as a smartphone (24, 25). Although the initial investment in the equipment may be considerable, many of these simulators can theoretically be reused indefinitely, making them more cost-effective in the long run. Enhancing the availability of 3D printing technology in low- and middle-income countries is a highly sustainable and effective strategy to expand global access to neurosurgical care. Dos Santos Rubio et al. report an illustrative case of a neurosurgical emergency in the Caribbean—a region with several differences in financial status, healthcare policies, and languages and characterized by challenging geographical conditions—to highlight the need to improve neurosurgical care and access by sharing tasks with the use of new technologies and networks (26). An expansive global network facilitates advanced training for neurosurgeons around the world and could empower surgeons in remote regions. By fostering the exchange of these cutting-edge technologies and expertise through accessible platforms, clinicians can gain fundamental neurosurgical skills, including burr hole evacuation, placement of the external ventricular shunt, and craniotomy, bridging the gap between regions with varying levels of medical infrastructure (26).

Through the assessment through the questionnaire, certain limitations of our model emerged, necessitating their resolution to facilitate further development progress. Although all respondents acknowledged the model's validity, we found some disagreements concerning the level of intraventricular details and the quality of tumor removal. This suggests room for improvement and technical improvements. Furthermore, the ventricular anatomy could be improved by adding crucial structures such as the choroid plexus and intraventricular vessels. In addition, this study is constrained by its relatively small sample size, which may impact the generalizability of the findings. Also, since some participants had never performed a real-life endoscopy before, their comparative evaluation of the model could be limited, especially the haptic perception, since there is a lack of comparison. The tutor's feedback was not evaluated, so rating the simulator from the teacher's perspective could add additional validation.

## 5 Conclusion

Our in-house designed, low-cost 3D-printed endoscopy simulator provides an accessible and practical training resource for neurosurgical residents, effectively improving their endoscope handling skills and coordination. Although it addresses the limitations of traditional training methods and contributes to procedural skill acquisition and competency, further advances are needed to improve and expand its application in training.

## Data Availability

The original contributions presented in the study are included in the article/Supplementary Material, further inquiries can be directed to the corresponding author/s.
