# Supplementary figures and images for: Innovating neurosurgical training: a comprehensive evaluation of a 3D-printed intraventricular neuroendoscopy simulator and systematic review of the literature
Source: Front Surg. 2024 Nov 5;11:1446067. doi: 10.3389/fsurg.2024.1446067 (PMC11573785; doi:10.3389/fsurg.2024.1446067)

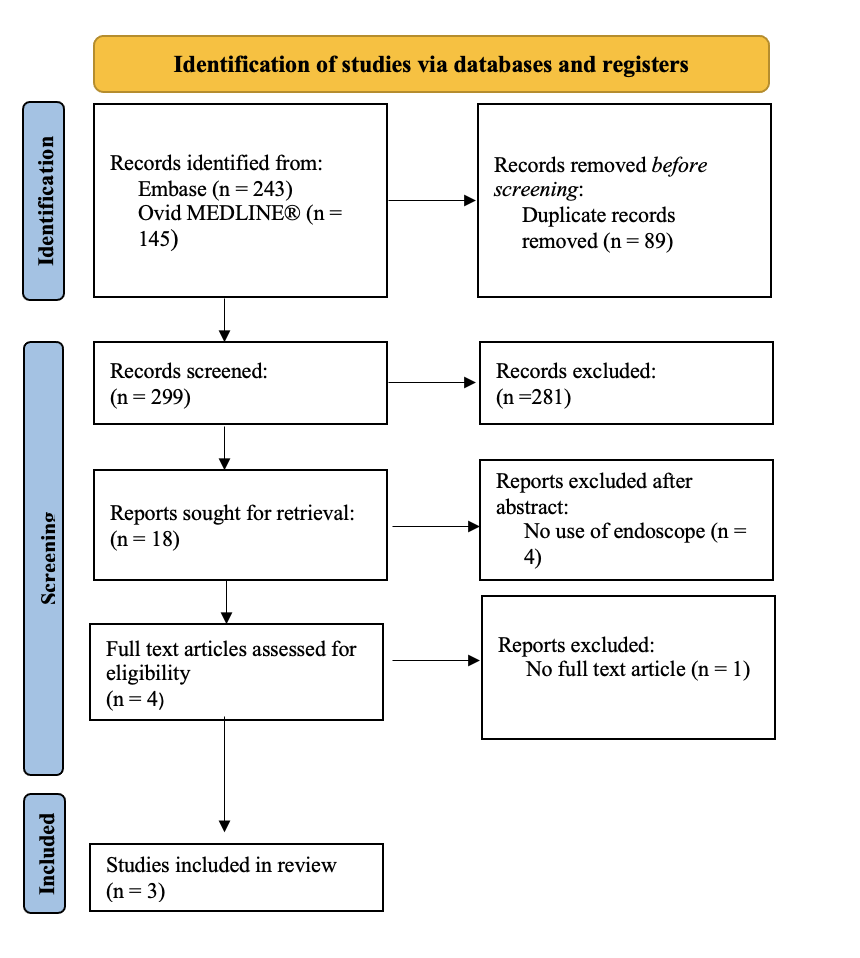


**Supplemental Figure 1.** PRISMA 2020 flow diagram for new systematic review

Supplement: Supplementary file 1 [file Datasheet1.docx]
